# "The Hospital" Nursing Mirror

**Published:** 1899-01-14

**Authors:** 


"The Hospital" Nursing Mirror.

Being the Nursing Section of "The Hospital."

[Contributions for this Section of "The Hospital" should be addressed to the Editor, The Hospital, 28 & 29, Southampton Street, Strand, London, W.C., and should have the word "Nursing" plainly written in left-hand top corner of the envelope.]

### News from the Nursing World.

### THE VICTORIA CROSS.

On the 6th inst. Her Majesty conferred the Victoria Cross on those who distinguished themselves for bravery in the recent Soudan campaign, and also conferred the order for distinguished service on Major Hugh Broderick Mathias, Royal Army Medical Staff, for service in the Nile Expedition of 1898. The ceremony took place at Osborne (where the Queen is now resident), and the Princess Henry of Battenberg, the Duchess of Albany, and the Princess Victoria were present on the occasion.

### OUR QUEEN AND HER PURDAH SUBJECTS.

The strict seclusion or "purdah" in which a large number of the women of India live, and which makes it impossible for them to permit a man to give them medical advice, causes an untold amount of misery. It is no matter of surprise, therefore, that our Queen should specially recommend Lady Dufferin's Fund to the lady who will officially represent her for the next five years. Lady Curzon, in promising to foster it, is only following in the footsteps of her predecessors, Lady Dufferin and Lady Elgin. The Fund was founded in 1884 by the former, in order to inaugurate hospitals and dispensaries for women and children, and to provide for the medical education of women for the purpose of enabling them to administer relief to the womankind of India. The Princess of Wales is patroness of a branch for collecting money for it in England; and, thanks to the encouragement of many eminent ladies, and also to the approval with which the object is regarded by the educated natives, high caste girls are coming forward in increasing numbers to be prepared for the work. The magnitude of the task is, however, incalculable. English lady doctors, who have been the pioneers of the movement, give details and figures that are simply appalling, and we trust that Lady Curzon's influence will help all women who are working against such fearful odds for the healing in sickness of our Queen's purdah subjects, and will greatly add to their number.

### FOR BRADFORD NURSES.

Mrs. Boyd Carpenter formally opened the new nurses' home at Bradford on Saturday last. A large number of guests were present, including the Bishop of Ripon, the Mayor and Mayoress of Bradford, Sir Francis Sharp Powell, M.P., and Mrs. Powell, and Sir W. C. and Mrs. Lupton. After vain efforts to buy the house in which the infirmary nurses have hitherto been lodged, the Guardians have had the present home—named the Victoria—designed and built for their accommodation. The cost has been £11,000, of which sum £9,000 have been already subscribed. An endeavour has been made to render the interior of the institution as comfortable as possible for those who will reside in it. The kitchens and caretaker's quarters are situated in the basement, the matron's apartments, sitting and visitors' room, and some bedrooms on the ground floor, the two floors above being devoted to sleeping accommodation and bath-rooms. In addition to the matron and night superintendent, there are 12 charge nurses and 38 probationers on the staff, and out of this number there are only two who have not been trained in the infirmary. Mrs. Boyd Carpenter was presented with an inscribed key, with which she opened the home, and said, in her little speech later in the proceedings, that she hoped the new building "would be a home of rest, peace, and refreshment to those whose lives were spent in alleviating the sufferings of their fellow human beings."

### FOR THE DUCHESS'S NURSES' FUND.

Chatsworth was the scene of two brilliant entertainments last week, the proceeds of which are to swell the Duchess of Devonshire's Nurses' Fund. The ballroom was arranged as a theatre for the representation of "A Fool's Paradise." Mr. Alexander Stuart, from the "Garrick," was stage manager, whilst special scenery was prepared for the first act. The ladies of high degree who took part in the performance were exquisitely dressed, and the acting was heartily applauded.

### ST. ALBANS DIOCESAN NURSES.

For 25 years the St. Albans Diocesan Institution for Trained Nurses has pursued the even tenor of its way. At one time it reached the zenith of its usefulness, employing some 40 nurses, but now so many institutions cover the same ground that a steady average of 23 is enough for its requirements. The staff when not on duty reside with the matron at Witham. The nurses have been employed in 80 parishes during the year, and are supplied at low fees —or even none at all—to the necessitous sick. The Claughton Convalescent Home at Walton-on-the-Naze, which is a useful adjunct to the work of the society, and is in the care of Miss Leader, the sister-in-charge, will shortly be permanently handed over to the institution free of debt.

### THE RUSH ABROAD.

The spirit of the age is restless, and our nurses have not escaped being possessed by it. Let a call come for them for Klondyke, for war, for plague, for South Africa, and hundreds respond with an eager "Send me"; and when the choice falls upon another a crowd of disappointed women surge through every door that seems to open to them some new experience. Would it were possible to believe that every individual of this throng was actuated by an unselfish desire to succour distressed humanity! But when an equally urgent summons is heard from country districts, from the uncongenial infirmary wards of the workhouse, from dozens of other dull places where the hardship is no greater, but where the voice of fame is silent, the cry, alas! is left unheeded. The known, the monotonous, and the comparatively safe offers no attraction to a vivid imagination, and ardent souls rush abroad to win, as they fondly hope, fame and fortune, to encounter perchance failure and ruin. English women are reared in what may be termed a garden of safety, hedged round by the chivalry of English men, and, because this is so, thoughtless girls arrogate to themselves a sceptre they deem universal. This misconception places them at the mercy of those men and women in whom a woman's helplessness arouses not chivalry but cupidity. We reiterate what we have urged times out of number—what after all is but the personal experience of those who know the facts—that no English woman, whether she be nurse, governess, or of other calling, should quit her country except for definite service to a well-recommended employ. Let her make sure first to whom she is going, and to what.

### A NURSE'S SUPERANNUATION.

Two points are instructive in the discussion of the Belper Guardians anent the superannuation allowance to be granted to Nurse Chawner upon her resignation. For 11 years Nurse Chawner has served the board, and she is now a confirmed invalid, having permanently injured her health in the discharge of her duties. She, therefore, sent in her resignation, applying for a superannuation allowance, and asking the board to exercise their prerogative of adding (in the words of the Act) a "period not exceeding ten years" to her length of service. When Mr. Marsden proposed that a period of four years be added to the 11 years of actual service the discussion upon the point became heated. One member "thought it cheeky on the nurse's part to have made the application"; another asked "if anyone could explain how Nurse Chawner had contracted her illness in the performance of her duties." Finally, by 10 to eight votes, this nurse obtained the desired recognition of four additional years of service, her allowance being thus calculated on the scale of £1 5s. for 15 years instead of eleven, or at the rate of £18 15s. a year for life. When it is clearly understood that this allowance is paid from a fund arbitrarily exacted from the salaries of the poor-law officers for this very purpose, the attitude of some members of the board appears in a very unpleasant light. Last week we gave an instance of an aged nurse "forgetting" how old she was, because Guardians, fearing that old nurses may come upon their superannuation allowance, will not engage them; this week we have the spectacle of a board wrangling over the allowance of an old servant broken down in health—two examples of the working of the Superannuation Act that cannot fail to condemn it in the eyes of all who have the interests of the nurse at heart.

### AN EXAMPLE TO FOLLOW.

The committee of the Kent and Canterbury Nurses' Institute have just made a most commendable resolution. Feeling the urgent importance of enabling their nurses to provide for their future they have adopted a scheme to affiliate with the Royal National Pension Fund for Nurses, and to afford their staff considerable help in the matter. This year, owing to unusual prosperity, the committee have also been able to distribute a bonus to their nurses, based upon the length of their services.

### NURSING AT WHITBY.

In March of last year a home was badly needed for the district nurses at Whitby, for the work of the association had during the two years of its existence outgrown the strength of one nurse. The absence of any provision in the town and neighbourhood for emergencies suggested the advisability of the committee taking a larger house than needed for the nurses and using it as a cottage hospital. This has been done, and very useful the combined institution has been during the past eight months; 29 cases have been nursed at the hospital, and 94 patients in the district. Both hospital and nurses have received liberal support from the residents, and the matron and nurse have won general approbation and regard for their energetic management and for the cheerful spirit in which they have faced many discomforts.

### NURSING THE NORFOLK POOR.

The Right Hon. Sir Nathaniel Lindley, Master of the Rolls, recently presided over an influential meeting held in the Royal Hotel, Norwich, to consider the desirability of forming a Cottage Nursing Federation. The aim of the meeting was to provide sick nurses for the poor, but no one seemed to know exactly what grade of nurse was desirable, and there was a tendency to appreciate the value of the cottage nurse. In October last Lady Leicester convened a meeting for a similar purpose, of which we have heard nothing more.

### SHORT ITEMS.

At the Isleworth Infirmary there is a Training School for Nurses. Probationers are received as vacancies occur. These enter for three years' training, receiving £5 for the first, £10 for the second, and £20 for the third year, with uniform. The nurses and probationers reside in a Nurses' Home, each one having a separate bedroom. Lectures are given by the medical staff, and examinations held as to efficiency. —Colonel W. W. Pilkington has promised £1,000 for the purchase of a new and more commodious house for the St. Helen's Mission Nurses.—Mrs. Archibald Little informs us that she has received a contribution to the "Natural Feet Society" of China in a roundabout fashion, and asks us to state that her address is 7, Park Place, St. James Street, S.W.—The decorations in honour of the Hospital Ball held recently at the Guildhall, Grantham, were allowed to remain for a second ball in aid of the Grantham District Nurse Fund.—The total sum raised by the supplementary Celtic Bazaar at Campeltown reached £340, greatly to the satisfaction of the Duchess of Argyll. Her Grace telegraphed her thanks to those who had helped.—Lady Zetland joined the guests at the Middlesbrough Nurses' Home on the occasion of the annual Christmas treat to 600 of the poorest children in the town. Her ladyship helped to distribute the fruit, sweetmeats, and toys with which the tree was laden.—The authorities of the Bulawayo Hospital have been flooded with applications for appointments from nurses in all parts of South Africa. At present the staff is limited to Miss Ronaldson, the matron, and about a dozen nurses, who, until the new home is ready, will be quartered in the private wards attached to the new hospital.—The board of the Town and District Hospital, Newark-on-Trent, have, with extreme regret, accepted the resignation of the matron, Miss F. Finlay, and have passed a resolution expressing their appreciation of her work during the last three years. Miss Finlay has been seriously ill, and is therefore giving up active work for the present.

### Hints on the Home Nursing of Sick Children.

By J. D. E. Mortimer, M.B., F.R.C.S., formerly Surgical Registrar, &c., at the Hospital for Sick Children, Great Ormond Street.

### PREFACE.

These "Hints" have been put together in the hope that they may be found useful by those who have already some acquaintance with the principles and practice of nursing in general, and who are called upon to take charge of a child during acute illness or in a serious stage of some chronic disorder. Without assuming the possession of such knowledge, it would, in fact, be impossible to keep these articles within bounds; and for a similar reason I have made no attempt to deal with such topics as the management and diet of the new born infant, the hygiene of the nursery, &c., which lie somewhat outside the limits of "nursing" in the strict sense of the term, and are also dealt with in numerous works, both elementary and elaborate.

It is not necessary to say much about those traits of character which are the

### Qualifications of a Good Children's Nurse.

Power of self-control, firmness without harshness, tidiness, promptitude, and so forth, are, of course, no less essential than in the case of grown-up patients. Children are, as a rule, singularly uncomplaining even under long-continued suffering; but at the same time it is requisite that the nurse should possess a cheerful, equable disposition, which will prevent her from being irritated by the fretfulness she may, perhaps, have to encounter, and also that, without mawkish sentimentality, she should have a genuine love and sympathy for little children. Nurses who do excellently with adults frequently fail in this last respect.

Nor need I insist at length on the extreme importance of carrying out the instructions of the doctor and of furnishing him with a full and accurate report. Whilst it is the nurse's first duty to record facts and not opinions, any remark or suggestion made by a judicious observer who has the advantage of being constantly with the patient is always worth careful consideration, and may be of the greatest value. I need hardly add that if from forgetfulness or insuperable difficulty any order has not been carried out, there should be no hesitation in reporting the circumstances.

### On Taking Charge of a sick child, besides the usual history of the illness, a stranger should, by inquiry and observation, obtain all possible information in regard to the disposition when in ordinary health, and, as far as may be without risk of offence, in regard to the previous training, whether the child has been habitually repressed and treated too strictly or spoilt by too much indulgence, and by being allowed to make a fuss about trifles. Even apparently trivial or exaggerated stories may be listened to if there is time, although the nurse should, of course, form her own opinion as to their real value.

Every baby and young child has little ways and even a language of its own, and a newcomer, unless provided with some key to the puzzle may meet with many difficulties, or even completely fail in her task.

It must be admitted that the strict routine of a hospital, which in its proper place is so indispensable and as a rule works so satisfactorily, does not help a trained nurse to encounter certain difficulties met with in home nursing, and it is here that her natural gifts come to the front. Whilst ever keeping in her mind a high ideal standard she must recognise the fact that in most households it is impossible to carry this out in every detail, and that she may best attain her end, *i.e.*, the welfare of her patient, by adapting her methods to circumstances and by wise concession in comparatively unimportant matters. She should not forget that, especially when the illness is believed to be dangerous, the relatives of any patient, not to say the parents of a sick child, are very often themselves, in a sense, ill from anxiety, and may consequently show some irritability or want of consideration, which should on no account be resented. Still less should a nurse convey the impression, by a supercilious or dictatorial manner, that she has come to supersede the relations or the usual attendants on account of their incompetence. It is, of course, true that in some cases those nearest akin to the little patient are so defective in the natural qualifications of a nurse, or so unstrung by distress or prolonged watching, that they are the last persons one would wish to see in the sick-room, but in such instances more may be gained, as a rule, by tact and persuasion than by laying down the law in a manner which is almost sure to provoke antagonism.

Unless the patient is in urgent need of assistance, it is not a good plan to go straight up to make acquaintance. Babies and even older children are apt to strongly resent being "rushed at." It is better for the nurse, without taking any direct notice of the patient at all, to talk quietly for a short time to the mother or someone else in the room, and if the child is likely to be interested some topic, such as the household pets, should be chosen. When left to herself the nurse should make gradual advances by arranging the toys or bedclothes and by saying a few words in a natural tone.

If the patient is asleep the nurse should keep out of sight. The sudden appearance of a stranger on waking may cause a serious fright.

A want of previous training in the formation of good habits may be a source of much trouble. Very tiny babies, who are accustomed to be fed at irregular intervals, to be constantly carried in the arms, to be invariably rocked or sung to sleep, will strongly object to more rational methods. The disturbance ceases when the expected indulgence is granted, but only for awhile, and it is a far kinder plan not to do so, but to let the baby "have its cry out" once or twice, when it will in all probability be found that the habit has been broken, and that henceforth there will be no crying except for legitimate reasons. In most cases this can be done without any harm resulting, but, on the contrary, very considerable benefit. The doctor's approval should, however, be first obtained.

Older children can generally be better managed by quiet assumption of authority, and by the employment of tact rather than severity. With them it may be found wiser not to deprive them too suddenly of indulgences to which they have long been accustomed, but there is no rule of universal application, and the individual character and probable effect of a line of treatment must be carefully considered in each case.

As regards the sick room general rules apply, but it must be remembered that children, with their small bodies and sensitive nervous systems, are very susceptible to cold. As Dr. Squire says, "if a child and thermometer are moved together to a cold place the child may suffer from chill before the thermometer has had time to show how low the temperature is." At the same time proper ventilation is of the first importance. There is never harm in an open window as long as there is no fog admitted, no cold draught playing on the bed, and no undue alteration in the general temperature of the room. Medicines, liniments, &c., must be put away in some safe place. In case of the responsible nurse having to leave anyone else in charge whilst she takes her necessary rest or recreation, the most careful instructions should be given and nothing be taken for granted—even the possession of what is usually called "common sense."

*(To be continued.)*

### Christmas Entertainments.

THE METROPOLITAN HOSPITAL, KINGSLAND ROAD.

THE Christmas treat to the patients of this hospital took the form of excellent programmes of music, song, and recitation on two successive evenings. The wisdom of fixing the hours from half-past four to half-past seven was obvious, and a number of patients, adults and children, were enabled to enjoy the really good music at a seasonable time. The wards looked very comfortable and festive, the chief feature of the decorations being the innumerable fairy lamps lent for the occasion by one of the hospital's best friends, Mr. Defries. Indeed, all of the "extras" were lent—the piano by Mr. John Brinsmead, the stage furniture by Mr. Fred. Hillier, whilst the programmes were the gift of Messrs. Eden Fisher and Co. There were no less than thirty-seven items on the first programme, which included a recitation from Rudyard Kipling by Mr. Lewis Waller, a flute solo by Mr. Albert Fransella, ventriloquism by Mr. Fred Russel. This hospital is so little known that it may not be out of place to call attention to its extreme poverty. "It is tragic" is the evidence of one of the sisters. "If anything delays the return of the linen from wash there are no sheets to make up a bed, and the doctor has no towel on which to dry his hands." Yet somehow or other the patients looked snug and comfortable, and the lights gleamed on the glossy leaves of the Christmas adornments. The babies had fallen asleep with their hands full of the rifled treasures of their Christmas tree, and only the sisters and those who have to pay the bills know how very tightly the shoe pinches.

ROYAL LONDON OPHTHALMIC HOSPITAL, MOORFIELDS.

THE Christmas entertainment to the patients at the Moorfields Eye Hospital is always a successful function. This year the programme consisted of a concert, on the result of which Miss Robinson and those who helped her to get it up are to be much congratulated. Miss Sybil Noble's singing, in character, of "Kentucky Babe" and "Wots the Use of Hannyfink," met with great applause, and the "Sketches" by "The Crocks," "The Pierrots," and "The Jonathan Johnnies," all hailing from the London Hospital, were excellent. "The Crocks" got themselves up alike (Messrs. Waldron, Smith, Williams, and Moritz) in grey hats, grey overcoats, and white gloves, the coats concealing other wonderful costumes which appeared later. The "London" men have certainly surpassed themselves this year, and their adaptations of familiar and popular songs, with many topical additions and allusions, were very clever, and thoroughly appreciated by their audience.

NORTH-EASTERN HOSPITAL FOR CHILDREN, HACKNEY ROAD, N.E.

THE Christmas entertainment to the little patients of this hospital was given on Bank Holiday. The day began with carol singing, which was followed by a distribution of toys, sweetmeats, and cards, when happily, through the kindness of friends, everyone received a share of these good things. After dinner, at mid-day, the time was occupied in the enjoyment of Punch and Judy, a magic lantern show, and all kinds of games. Tuesday was also spent in merry-making, and on the 11th inst. Christmas trees will be the order of the day, and are already eagerly anticipated.

MEMORIAL HOSPITAL, MILDMAY PARK, LONDON, N.

THE Christmas festivities here began on Christmas Eve with the usual dinner of turkeys, plum pudding, and dessert. The wards were very tastefully decorated by the patients and nurses; the latter sang some solos and hymns during the afternoon, and again at seven o'clock on Sunday morning. Between the usual morning and evening ward services the patients were greatly pleased listening to some Christmas carols which the choir of St. Paul's, Canonbury, very kindly came and sang in the hospital. The annual tea and a service of song, with some music and recitations, took place on the following Tuesday, when an evening which everyone seemed thoroughly to enjoy was brought to a close by the ever welcome appearance of Father Christmas in full costume, distributing the gifts of clothing to the patients which Lady Hay, Miss Harward, and other kind friends had sent.

ISLEWORTH INFIRMARY.

THE sisters and nurses of this infirmary took much pains in making the wards and patients smart for the Christmastide festival. The babies wore muslin pinafores tied with scarlet ribbon in honour of the occasion, and *Truth's* silver sixpences hung round several necks, whilst a doll as big as themselves, gorgeous in pink satin—also one of *Truth's* gifts—filled them with an awe of delight. Good fare was provided in all departments, and a musical and dramatic programme, which occupied the afternoon, was given by friends of the medical superintendent, to the great pleasure of all able to be present. A novel feature in the decoration was the use of numerous fans, which led to one ward being dubbed "Fanity Fair."

NEWTON ABBOT WORKHOUSE.

ALL the world heard of the troubles at Newton Abbot Workhouse some five years ago, and a correspondent thinks it only fair that it should know of the happier state of present affairs. An account of the Christmastide in the new infirmary answers this purpose excellently. Evergreens, mottoes, lanterns, and flowers were all pressed into the service to make the wards festive in appearance. An ample supply of seasonable fare was provided, at the distribution of which several of the Guardians assisted personally, and one of them, Dr. Fey, gave a lantern entertainment in the evening. On the 2nd inst. the New Year treat consisted of a sumptuous tea, after which appropriate gifts of tea and sugar to the ladies, tobacco for the men, and toys for the children were presented, whilst the evening was occupied with a concert, during an interval of which light refreshments in the shape of cake, nuts, and oranges were handed round.

### Nurses for Workhouse.

SOME little time ago we noticed that the Runcorn Guardians and those of Yarmouth had petitioned the Local Government Board upon the subject of providing facilities for training nurses for employment in their workhouses, and Miss Louisa Twining, as the mouthpiece of the Workhouse Nursing Association, suggests the following remedies for the existing deadlock in the current number of the *Municipal Journal*, of which we give a summary:—

1. Every poor law infirmary or workhouse infirmary wards of over 200 beds to be utilised for training probationers on the understanding that they remain in the service of the poor law. The State to guarantee pension at the end of a certain period of service. 2. The nurse to be free from the interference in nursing matters of the master and matron, and to be directly responsible to the medical officer only. 3. Salaries to be higher and accommodation more comfortable. Expenses of training to be borne by the State. 4. That guardians should form themselves into a central committee or into local and county committees, and thus bring greater weight and authority to bear on the Central Board, that is, the Local Government Board, and 5. That this committee or committees should unite with the Central Committee of Poor Law Conferences.

### Training in the Provinces.

(*Continued from page* 129.)

### NORTH STAFFORDSHIRE INFIRMARY AND EYE HOSPITAL (216 Beds).

### Terms of Training.

Candidates for training at the North Staffordshire Infirmary must be between 21 and 32 years of age, and "well educated." If accepted at the end of a month's trial they are required to enter into a year's engagement as probationers; if found to be efficient they are then expected to remain in the service of the hospital as probationer nurses for two years longer. No premium is required, as the training extends over three years, and salary begins at £10 for the first year, rising to £12 for the second, and £15 for the third. Those nurses who wish to remain after the expiration of their three years, and are considered suitable by the matron, receive a salary of £20. The charge nurses' places are filled up as vacancies occur, as far as possible, from amongst the most suitable of the probationer nurses who have completed their training. Charge nurses' salaries range from a minimum of £25 to a maximum of £30, according to the importance of the post and length of service. Any probationer breaking her agreement, except on a certificate of illness or inability from the Medical Board, is required to give three months' notice and pay a penalty of £5.

Night duty is taken by second and third year probationers, working under a night superintendent, for from three to six months at a time.

### Hours On and Off Duty.

Probationers are on duty in the wards between 7 a.m. and 9 p.m.. deducting two hours daily for recreation, and time for meals. The two hours are either from 2 to 4, 5 to 7, or 7 to 9 p.m. Charge nurses are on duty from 7.55 a.m. to 9 p.m. In addition to the daily two hours' recreation they have leave from 3 to 9.30 p.m. once a week. Probationers have similar leave once a fortnight, and all the staff are off duty on alternate Sundays from 9 a.m. to 1 p.m., or from 5 to 9.30 p.m. Night nurses' hours on duty are from 8.55 p.m. to 8 a.m., with two and a half hours daily for exercise. Nurses and probationers have 14 days' yearly leave of absence; charge nurses, 21 days.

### Meals.

All meals, except the early morning luncheon and the midnight tea, are served in the general mess-room. Lunch is taken in the ward kitchens, and it is for these meals only that allowances of coffee and sugar are made. The day probationers breakfast at 6.40 a.m.,; dinner is at 1.30 p.m., tea at 4, and supper at 9 p.m. Charge nurses breakfast at 7.30; their dinner is at 1 p.m., tea at 4.30, and supper at 9 p.m. The night nurses have their breakfast at 8.30 p.m., and their dinner at 9 a.m.

### SUNDERLAND INFIRMARY (214 Beds).

### Terms of Training.

Probationers are received for three years' training at the Sunderland Infirmary, the age considered desirable being from 21 to 32. A certificate is given on the satisfactory completion of the three years' engagement, lectures being attended during the training and prizes awarded after each course. No premium is required, salary beginning the first year at £10, and rising to £16 and then £18. Staff nurses' salaries range from £22 to £26, charge nurses receiving £30 per annum. The position of sister is an honorary one, and is filled by deaconesses from the Deaconesses' Institution and Hospital, Tottenham. Charge nurses are chosen from the infirmary staff, a three years' certificate being an essential qualification. They are further selected for administrative ability, education, tact, refinement, at the discretion of the Managing Sister. Laundry and indoor uniform are provided by the hospital. Night duty is taken for three months in each year by all nurses and probationers. The work of night superintendent is taken in turn by charge nurses, this arrangement being considered better for the nurses.

### Hours On and Off Duty.

The day nurses' hours on duty are from 7.30 a.m. to 9 p.m., but at 8.30 p.m. they are relieved for supper. Time for lunch, dinner, and tea must be deducted from these hours. Probationers during their first six months have two hours off duty every day, and by special arrangement the nurses in the children's wards have leave each day, from 3 to 5 one day and from 6.30 p.m. for the rest of the day in alternation. The rest of the day staff are off duty every other day from 3 to 5 p.m. Sisters and charge nurses' hours are the same, with the addition of two free evenings in the week after 7 p.m. No regular days off are arranged for. They are given when requested by the nurses as may be found possible. Night nurses are on duty from 9 p.m. to 7.30 a.m.

### Meals.

All meals are served in the dining-room, and with the exception of the informal early lunch, are presided over by a sister. No weekly "allowances" are given to the nurses, everything being amply provided for each meal, as at a private family table, and no meals, either night or day, are allowed in ward kitchens. The hours of meals for the day nurses and probationers are as follows: Breakfast, 6.45 a.m.; lunch, 10.30; dinner, 2.15; tea, 5; and supper, 8.30 p.m. The sisters and charge nurses have their meals in a separate dining-room half an hour earlier, with the exception of breakfast, which is at the same hour as that of the rest of the staff.

### To Pension Fund Nurses.

### MISS BURNS' WEDDING GIFT.

We publish to-day the final list of contributors to the presentation of a congratulatory address to Miss Burns, the daughter of the late Chairman of the Pension Fund, on her forthcoming marriage. We shall cause the address to be prepared, and when it is ready each contributor will receive an invitation to view it, together with a financial statement. The letters we have received prove that the devotion to their interests displayed by their late chairman has not passed unrecognised by Pension Fund Nurses. We take this opportunity of thanking many writers for the very kind words of appreciation towards ourselves which they have expressed. H. E. Doughty, Policy 3,988, M. D. Ewing, M. A. Dibley, Policy 2,119, E. Rutherford, E. S. Sutchell, E. K. Philp, M. D. Little, M. A. Plowman, H. M. Hill, F. L. J. King, B. J. Davies, E. A. Wilson, E. E. Thomson, H. Robatham, M. E. Tatt, C. B. E., A. E. Briggs, F. K. Gardner, E. Ratcliffe, J. Campbell, L. J. Attree, Two Gloucester Nurses, Nurse Headford.

### Appointments.

Ulster Hospital for Women and Children, Belfast.—Miss Harriet A. G. Fraser, who was trained at the Birmingham General Hospital, and at the Royal Maternity Hospital, Edinburgh, was appointed Matron here on January 5th. She became staff nurse and sister at her training school, and was afterwards night superintendent at St. Mary Abbotts Infirmary, Kensington. She has since been attached to H.R.H. Princess Christian's Trained Nurses' Home, Windsor.

### Nursing in Paris Hospitals.

### C.—THE NURSING SISTERS.

### IX.—Their Present Domains—The Hôtel Dieu and Saint Louis.

And now I must conclude my account of the Sisters and of the Paris nurses generally by coming back to their ancient homes, the Hôtel Dieu, mother house of all the nursing establishments, and its eldest child, the great Saint Louis Hospital.

Outwardly there is nothing ancient or venerable about the Hôtel Dieu of to-day. It is only the traditions and the situation which are ancient. Even the situation is not, mathematically speaking, of lineage from the past. The Parisian ediles have chosen to shove the new hospital a few rods to the northward, and before long the sole remaining wing of the old structure on the southern bank will be demolished, and the last link severed with the adjoining curious little old church of Saint Julian-the-Poor, for ages the chapel of the Hôtel Dieu nurses, and now given up to the handful of Parisian Greeks who chose to attend their gorgeous national religious observances. Still the new Hôtel Dieu faces on the Place du Parvis Notre Dame, the corcordium of ancient historical Paris, and this savours enough of antiquity. Unfortunately, with all its modern changes of front as well as place, the new hospital is not a very creditable affair. Rather handsome and imposing without, it is bleak and ill-arranged within, and wasteful of space and opportunity, where both are so precious in this crowded heart of the city.

On the other hand, the Saint Louis Hospital combines the flavour of antiquity with many modern improvements. Situated of old in the suburbs, it is now in an out district, and has ample room. Although its buildings by no means date from the early days of the institution, they come down from three centuries ago, and their mansard gables and angles are some of the most delightful architectural joys of Paris. The Saint Louis Hospital is one of the largest, if not the largest hospital in the world, and one of the most important in many ways. Over these two great institutions, in spite of all the administrative storms of the last twenty years, the nursing Sisters still reign supreme. In each hospital I have had the privilege of long interviews with the Mother Superior, and in both cases I have found women with a full sense of responsibility of the present delicate situation, determined to hold what they consider their own domain, but to hold it by every reasonable concession to the demands of modern hospital ideas, and with a firm grip of all the details of the important task they are superintending.

At Saint Louis, especially, I discussed these details of the work with the Mother Superior, and found many effective retorts to the damaging allegations hurled at the Sisters' heads for the last twenty years during the laicisation compaign. Thus I found that in the staff of nurses at Saint Louis were included nine novices, being carefully trained in the hospital duties, and destined to become experienced matrons. Yet again and again has it been charged against the Sisters that they have no special training, and that it is considered a sin for novices to be exposed to any public gaze, so that all effective training in hospital work is impossible. All I can say is that such charges become ridiculous to anyone who takes the trouble to visit the great skin hospital of Paris, where the novices, just like the regular Sisters, will be seen quietly passing back and forth through the many courts and shaded walks, and up and down the sta rways and through the wards, in their picturesque attire, or in their working frocks, as the case may be. It would be indeed a prurient mind which, in that vast assemblage of affliction and pain and deformity, would find place for any thought of worldly levity or impropriety.

A more reasonable charge has been made against the Saint Louis Sisters, especially by Dr. Bourneville, of putting hindrances and petty annoyances in the way of lay nurses wishing to attend the neighbouring school at Lariboisière. I think we may take it that these episodes, if any, are only the minor inevitable misunderstandings or malicious differences sure to be raised in such an immense establishment as that of Saint Louis, where the employés know that two such antagonistic forces are to be appealed to in the administration above them, as the partisans of laics and Sisters must ever be. We may take it for granted that if any really serious attempt had been made by the Sisters to interfere with the full exercise of the right of the laics to attend the school the strong hand of M. Peyron would long since have come down with crushing effect upon them. Although M. Peyron is no longer in office, having been replaced in May last, his successor, Dr. Napias, is not likely to be a less vigilant protector of laic rights.

In summing up the position to-day and the ground still held by the Sisters, we must not compute mere numbers. The Hôtel Dieu by its prestige counts for an immense force, but after all, in a purely pathological way, it is one of the ordinary hospitals. On the other hand, Saint Louis is far and away not only the most important hospital, but of importance in comparison far beyond even its great extent. M. du Camp, in his work before quoted, a generation ago expatiated eloquently on the model appliances, especially the elaborate installations of medical baths at Saint Louis, and the great improvements and discoveries made in skin disease treatment, from Thierry downward, during the last hundred years. Of course, all the credit of Saint Louis must redound in a certain extent to the chief nurses who have to carry out the details of the work. These chiefs have ever been and are to-day the pious Sisters.

Again, in summing up their domain, the women's prison of Saint Lazare, which I have mentioned, must also be reckoned, for Saint Lazare is, after all, largely a hospital; and, moreover, at Saint Lazare there is one incidental feature which is an answer to one of Dr. Bourneville's most venomous charges. The Sisters are in charge of the pharmacy there, and I do not believe they simply sort the drugs into "blue and green, &c.," or my friend, M. Maugé, the director of the prison, would soon interfere.

Finally, as to the future. He is a bold prophet who prophesies anything in France even for the morrow. All things are so interwoven. Even the inevitable Dreyfus case may have its effect on the laicisation question. Unfortunately, certain portions of the clerical and monarchical elements have played a very shabby and certainly a losing part in this performance, and it may be used as a lever for attack on everything religious all along the line, even against the virtuous Sisters. Let us hope that a spirit of enlightenment will rise to see the folly of attacking poor nuns working in humble benevolence because political Churchmen plot mischief. In fact, it is the very spirit of unscrupulous attack on the one side which breeds unscrupulous attacks on the other. I would finally advance with caution, as the probable outcome of the present situation in the nursing of the Paris hospitals that, though a large portion of the hospitals will remain in laic hands, the Sisters' domain will be gradually extended to a considerable degree. Edmund R. Spearman.

### Puddings for Invalids.

As a rule these have not much variety about them, and patients soon tire of the milk pudding, which perhaps is spoilt by being made with eggs, or the steamed custard that has little flavour about it. The following recipes will doubtless be of great use to many of our readers. Some are new, and all are composed of ingredients that can be easily digested.

Veroni Pudding.—Veroni being a preparation made by blending certain wheats rich in gluten and containing the maximum of albuminoids, it is especially suitable for invalids. It can be bought at any grocers. To make a small pudding, boil one pint of milk, stir into it one ounce of veroni and a tablespoonful of sugar; continue to cook it for ten minutes; beat two eggs well, and add these to the pudding with one ounce of butter and a few drops of vanilla essence. Pour the mixture into a buttered pie-dish, and bake in a moderate oven for half an hour.

Tapioca Pudding.—When fruit is allowed, this is a delicious pudding, and always liked. Cover the bottom of a pie dish with tapioca, and half fill it with cold water. Let this soak for one hour. Then nearly fill the dish with any fresh fruit, such as gooseberries, currants, raspberries, cherries, &c., and sprinkle them with sugar, and add more water. Bake in a moderate oven for an hour or so, according to size of the pudding, stirring it occasionally and adding more water if it gets dry. When the tapioca is quite clear and fruit cooked, make a meringue by beating together the white of an egg and tablespoonful of castor sugar. Put this on the top of the pudding, and return it to the oven for a few minutes, when it should be a light brown.

Sago Souffle.—The small bird-seed sago is the nicest and best to use; well wash three tablespoonfuls, and boil the sago in half a pint of milk; when cooked and clear, sweeten with a teaspoonful of sugar, and stir in the yolks of two eggs. Let the mixture cool; in the meantime beat two whites of eggs stiffly with a pinch of salt, mix these in very lightly, pour the soufflé into a buttered dish (soufflé), surround it with a buttered band of paper, tie it, and bake it in a moderate oven for half an hour, take up, remove the paper, put a folded d'oyley round, and serve as quickly as possible.

A Simple Trifle.—Put on the stove in a saucepan half a pint of milk, as soon as it boils stir in quickly a tablespoonful of ground rice, keep it free from lumps, and cook till it is rather thick, add a few drops of vanilla essence, a teaspoonful of sugar, and the yolk of an egg; simmer for a few minutes, and when cool pour it into a glass dish. Allow to get quite cold, then spread a little jam or fresh fruit compôte on the top, and over this pour a custard or a little whipped cream, sweetened and flavoured with a few drops of vanilla.

A New Rice Pudding.—Blanch one and a half ounces of Carolina rice by putting it in a small saucepan, and covering it with cold water. As soon as the water comes to the boil wash the rice in cold water till the water is clear. Boil half a pint of milk, and in this cook the rice till it is tender. Then add an ounce and a half of sugar, and the same of finely-chopped suet, one whole egg well beaten, and a few drops of vanilla essence. This pudding should be quite moist. Put it in a well-buttered pie dish, and bake in a moderate oven till a light brown colour.

Brown Bread Cream.—Whip a quarter of a pint of double cream till it is stiff, then add a little sugar, a few drops of vanilla essence, and a teaspoonful of best brandy, with some brown bread crumbs rubbed through a coarse wire sieve, and stir a quarter of a pound of them into the whipped cream. This must be done carefully. Pile the cream in a glass dish and strew it with glacé cherries cut in shreds, or any fresh fruit that has been dipped in a little syrup or liqueur.

Custard to Serve With Fruit. — Boil half a pint of milk with two ounces of lump sugar and a small piece of lemon peel, mix one table-spoonful of cornflour with half a gill of cold milk, add it to the boiled milk, and let it cook for three minutes, stirring it all the time. Beat the yolks of three eggs in a basin and pour the hot mixture on to them; mix well and return all to the saucepan, stirring it over the fire till it thickens. Do not let it boil or it will curdle. Strain it into glasses when cold, and sprinkle on the top a few crushed ratafia biscuits.

Maccaroni Pudding.—Boil an ounce and a half of maccaroni in half a pint of milk, with a small strip of lemon peel. It should only simmer gently for a little more than half an hour. When quite cooked and tender add two eggs beaten up with another half pint of milk, a little sugar and nutmeg, and, if liked, a tablespoonful of brandy. Pour the pudding into a buttered pie dish, stand the dish in a tin containing boiling water, and bake it in very moderate oven for half an hour. If too hot the pudding will curdle.

Veroni-Vermicelli Pudding.—Put half a pint of milk on to boil in a saucepan, then stir into it when boiling an ounce and a half of veroni-vermicelli. Stir it well and cook till the vermicelli is tender, then add one ounce of butter, a little sugar, and one egg well beaten. Put it into a buttered pie dish, stand this in a tin containing boiling water, and bake for 15 minutes in a moderate oven.

Sponge Pudding.—Scald a pint of new milk, and let it get cold. Beat two ounces of butter and two of sugar together till they are creamy, add two ounces of flour; mix these with the cold milk and add three yolks of eggs well beaten, and the whites whipped stiffly with a pinch of salt. Put the mixture into a buttered pie dish. Stand the dish in a tin containing boiling water, and bake it for about half an hour in a moderate oven till it is a light brown colour.

Lemon Pudding.—This is nicest when eaten cold. Mix three tablespoonfuls of cornflour with cold water; then pour on to it three coffee cups of boiling water, and boil till it thickens; then add two cups of sugar, the grated rind and strained juice of two lemons, and two eggs well beaten and a little salt. Butter a pie dish and pour the mixture into it. Stand it in a tin containing boiling water, and bake it for 20 minutes. Serve a little cream with it when cold.

### The Humours of Hospital Life.

FOLLOWING on an article on the "Humours of Clerical Life," the *Cornhill Magazine* has published a yet more entertaining account of some "Humours of Hospital Life," from which we append the following anecdotes. If any class of human beings see human nature as it really is—see their fellow men and women at their best and at their worst, without the varnish of conventionality—surely hospital nurses have unrivalled opportunities for this study, though most of them are too busy or too tired to record their impressions, remarks the writer of this article, with assuredly much wisdom on her side.

After twelve years' experience of patients of every grade' the writer goes on to say that the callousness of the poorer classes with regard to sickness and death has by no means been exaggerated in the scenes from clerical life above referred to. "Though kind and helpful, as this class undoubtedly are to each other, yet their feelings get blunted by great familiarity and close contact with every form of suffering and disease," and the following stories will illustrate the condition:—

A hospital sister summoned the wife of one of her patients into her private room, and began to tell the woman gently that the doctors thought very gravely of her husband" case. "Well, Miss, that's jes wot I sez to 'im lawst visitin' day. Tom, I sez, I think you're breaking up, I sez. But we'd miss yer wages of a Saturday, I sez, if so be as it pleased the Lord to taike yer."

Another woman, summoned to see her dying husband, who was the victim of a street accident, showed her grief very plainly, throwing herself on the floor, and giving vent to effusive lamentations as her husband breathed his last. Quite shortly afterwards the woman came back to the hospital ward dressed in the deepest of widow's weeds. "Please, I've come for pore Walter's clothes," she said. "The Lord's took 'im, but I 'ope, please God, as I'll find another."

Much in the same strain the writer instances another woman, who, *àpropos* of her husband's death, observed: "Deed, aye, Tom's deid. The wee-est thing pits me aboot, ye ken."

Several stories are told about hospital chaplains. No doubt there are many earnest men who fill this difficult position with comfort to their sick parishioners and honour to themselves. But there are others who are lamentably devoid of that most essential of all virtues, the gift of tact. "Some medical students," according to our authority, "once averred that before appointing a chaplain, the committee must have advertised thus: 'Wanted, a person of limited intelligence and the plainest possible appearance to officiate as hospital chaplain. Terms very moderate.'"

But, having illustrated the intercourse between patient and chaplain, the writer passes on to the attitude of the patient to his doctor. As a rule the patient looks up to his medical attendant, especially to a visiting surgeon or physician, with implicit confidence and a good deal of wholesome awe and reverence, his anxiety, as the writer urges, being sometimes unintentionally comic in the way he tries to help the doctor.

A senior surgeon, it is said, was lecturing to a class of students on different appearances of the teeth. "Here, gentlemen, in these two teeth we have well-marked symptoms of ——" Patient (interrupting in a deprecating manner): "But, please, sir, them two's false 'uns." Now and then the doctor is believed to be almost omniscient. A patient in a military hospital was constantly getting into difficulties on account of food which he smuggled into the wards. One morning his medical officer was about to examine his throat with a laryngoscope. Seeing the little mirror all ready for use, the man's chum whispered an anxious warning from the adjoining bed, "I say, Bill, you'd best 'ave a care. 'Ee moight 'appen to see wot yer 'ad for supper lawst noight." On recovery the patient's gratitude to the doctor sometimes overflows in speeches like the following remark made by a poor woman after a long illness: "I wouldn't never 'ave got over my lawst illness if it 'adn't a bin for Surgeon-Captain Jones and the Lord." One other instance of gratitude we quote from the quaint reminiscences given in this article on a humorous side of life within hospital walls. An old man, who had been tended during his illness by a stately probationer, the daughter of a bishop, lay watching her clean some glasses near his bed. "W'en I gets out o' 'ere, my dear," he said, "I don't mind if I finds yer a nice comfortable sittivation as barmaid down 'Ackney way. You know 'ow to clean glass, and 'd better get money, anyhow."

### An Opening for Older Nurses: Lecturing.

WITHIN the last few years lectures on the nursing of the sick have been added to the syllabus of lectures of County Councils, School Boards, and local associations engaged in disseminating knowledge amongst the people. Hitherto many of the lecturers have been lay women coached rather than trained for the purpose of delivering the lectures. This is due to the fact that the number of candidates amongst trained nurses willing to give up their career and take to lecturing has been small. The demand for these lectures becomes larger every quarter, and the standard of efficiency demanded we are glad to say is rising. The value of a lecturer on any practical subject depends not only on the facility for fluent discourse, but, above all, upon the groundwork of practical knowledge which enables the lecturer to meet the queries that arise after a demonstration, and to elucidate the points which may not have been clear to all amongst the audience. The field is waiting for the workers, and the workers are willing to be employed. It remains only to bring the two together. We have been consulted in the matter, and asked to lend a helping hand. With this object in view we propose to bring together the trained nurse lecturer with those who require her services. We now invite those nurses who are already proficient lecturers, and also those who desire to be prepared as such, to communicate with us; the former with a view of being placed upon our list of lecturers, and the latter that we may assist them to obtain the necessary training to fit them to become lecturers. Letters marked "Lecturing" should be addressed to the Manager, Scientific Press, 28, Southampton Street, Strand, W.C. The middle-aged nurse is finding it increasingly difficult to maintain her footing in the field of active nursing. She may be both able in body and in mind, but her age is against her. Now, given that she is a woman of fair education, not insurmountably afflicted with the shyness which shuns the publicity of a platform, the middle-aged nurse should prove a very suitable person to lecture on her own subject. This is especially true in the case of a district nurse. No one knows better than she the hints most useful, for she has observed the errors made in the homes she has visited.

### OUR CONVALESCENT FUND.

SUBSCRIPTIONS to the above fund come dropping in very cheeringly, and we gladly acknowledge this week Miss L. J. Atten's gift of 5s. The reserve in our exchequer is, however, still too low to be regarded with anything like satisfaction.

### The Problem at Camberwell.

The squibs and crackers of journalism that have exploded beneath the sensational headings of "Cigarettes," "Rats," "Petty Tyranny," and the like, give no idea of the causes of the troubles—nursing and otherwise—at Camberwell. The root of the whole matter is that the Guardians are confronted by an onerous task, the magnitude of which no one but those intimately acquainted with the facts can have any comprehension. In the first place, in an infirmary of about three hundred beds accommodation is needed for some nine hundred cases, consequently the wards are full—as in a general hospital—of acute cases. As soon as a patient can go he has to be sent away, not because he is really well, but because his bed is needed for someone worse than himself. Then comes the question of nursing. The "cigarette" episode undoubtedly did considerable harm to the institution, as it kept away the right type of nurse, and Camberwell Infirmary not being a training school for nurses (and for this reason the authorities being unable to train their own probationers), they were more or less at the mercy of what may be called "detached" nurses, that is, women who had had a few months' experience in this or that home or fever hospital, but who were unacquainted with the exigencies of Poor-law requirements. Thus the really good nurses on the staff felt unable to cope with the heavy work, and the assistant nurse of the right kind went, after a short stay, where she cou'd earn a three year certificate. The reason that the nursing staff was not larger, and therefore better able to do the work, is the same that caused the barely convalescent patient to be turned away, namely, accommodation could not be obtained for them. It is easy to be seen from these facts that it was no question of engaging a few more nurses—the medical superintendent is authorised by the committee to engage as many well-trained nurses as he requires for the well-being of the patients—of recreation rooms and pianos (though these are now added), nor of the enforcement of such rules as obtain in well-disciplined institutions that has caused the ebb and flow of nurses at the infirmary. It is one of providing accommodation for nearly twice as many more patients as are housed at present, and of a sufficient number of nurses to wait upon them, that is, a staff of at least a hundred. Such provision is not made in a day, but the Guardians hope in the course of the next two years to own an infirmary and a nurses' home second to none. Towards this they have acquired an excellent spacious site adjoining the present building. The old workhouse and the other houses standing upon it will be razed to the ground, and the new erections will be in accordance with the best scientific requirements. The cost will be somewhere about £160,000.

So much for what is to be done in the future. What is now in progress is that the Local Government Board has been approached for the purpose of obtaining the recognition of the infirmary as a training school for nurses, and a communication is expected daily from them upon the subject. When the new arrangements are made it is proposed to place each group of wards, containing from 50 to 60 beds, under a hospital-trained charge nurse, two staff nurses, and two probationers.

During the year 46—not 56 as reported—nurses have resigned or been dismissed. The following are some of the reasons for this: Marriage, three; to acquire certificates, three; for better posts, three; for health reasons, four; on account of disagreement with fellow nurses, four; five were dismissed for incompetence; one for drunkenness; and two resigned because they considered the last dismissal unjust. Not one has complained of her treatment by the medical staff or matron. The latter, although untrained, has been a very good friend to the nurses, catering in a motherly fashion for their comfort, and exercising no jurisdiction whatever over their nursing duties, whilst the day and night superintendents have full control over their own departments under the medical superintendent. Neither has there been any complaint of "rats" and "petty tyranny." The heaviness of the work, the difficulty of getting good ready-trained nurses are valid and sufficient reasons for all the troubles of the nurses; these remain for the time being, nevertheless it is safe to predict that at the present moment young, strong, capable, well-trained nurses, who are desirous of showing the stuff of which they are made, have an excellent opportunity of coming to the front at Camberwell. With the new arrangements and increased staff good posts will be opened, and if there are discomforts to be faced and arduous duties to be performed, well, the nurse who rises to the occasion will as certainly be marked for promotion.

### Minor Appointments.

Royal Cornwall Infirmary, Truro.—On December 8th, Miss Emily E. Bradshaw, who was at the Crumpsall Infirmary, Manchester, and at St. George's Hospital, London, was appointed Charge Nurse here. Since then she has been engaged in district nursing at Barry, Cardiff, and plague nursing in India. On the same date Miss Frances Leng was also appointed Charge Nurse. She was trained at Southampton, and has been head nurse at the Royal Hospital, Richmond.

Shoreditch Infirmary, Hoxton Street.—Miss Annie Eafton, who was trained at the Manchester Royal Infirmary, has been appointed Night Superintendent of this infirmary. She has been staff nurse at the Barnes Convalescent Hospital, Cheadle; private and district nurse on the staff of the Oldham Nursing Association; and for the last two years head nurse at the Greenwich Union Infirmary.

Kettering Workhouse Infirmary.—Miss Fanny Elizabeth Bloxham has been appointed Superintendent Nurse of this infirmary. She was trained at the Crumpsall Workhouse Infirmary, Manchester. She has since worked in the Mildmay Nurses' Home, and has been sister at Crumpsall Workhouse Infirmary.

Infirmary of Training Ship "Shaftesbury."—On December 23rd Miss Louisa Wiskin was appointed Charge Nurse here. Miss Wiskin was trained at Guy's Hospital, where she remained for seven years. She afterwards took charge of the cottage hospital at Herne Bay for five years, and since then she has been engaged in private nursing.

Cottage Hospital, East Grinstead.—Miss Blanche Sleap has been appointed Matron here. She was trained at Guy's Hospital, and is at present matron of the Village Hospital, Buckhurst Hill, which post she has held for three years.

### Novelties for Nurses.

### SPECIALITIES FOR THE TOILETTE.

Messrs. J. Crosfield and Sons, of Warrington, have become formidable rivals to other manufacturers of toilette commodities. Their "Erasmic" herb specialities are most fragrant and pleasant, and the scent, soap, and face powder will form a welcome addition to the toilette. This year the firm have issued a Christmas annual, and with it a very pretty presentation plate. Both annual and plate can bear comparison with the best publications of the kind which have appeared this season. The annual is devoted to stories from the pens of Guy Boothby, Grant Allen, George Sims, and Edric Vredenburg. The illustrations are most artistic and numerous.

### For Reading to the Sick.

### RESTFULNESS.

### Verses.

BRIGHT comes the morn, and soft descends the night
On the fair dwelling-place of love and peace;
And from the buffetings of this rude world
Its happy inmates, like the wandering dove,
Hence to the ark for refuge there can fly.
Prayer meets no hindrance there; and praise from thence
Of hearts and lips in unison ascends
More acceptable to the God of Love.
The idol Self is from his throne cast down,
And God set up instead; and where He reigns
There must be happiness, there must be heaven. —*Lyte.*

The Father portioneth as He will
To all His beloved children, and shall we not be still?
Is not His will the wisest? is not His choice the best?
And in perfect acquiescence, is there not perfect rest!
—*F. R. Havergal.*

When the Rest of Faith is ended, and the Rest in Hope is past,
The Rest of Love remaineth, Sabbath of Life at last.
No more fleeting hours, hurrying down the day,
But golden stillness of glory, never to pass away!

Time, with its pressure of moments, mocking us as they fell,
With relentless beat of a footstep, hour by hour the knell
Of a hope or an aspiration, then shall have passed away,
Leaving a grand, calm leisure, leisure of endless day!
—*F. R. Havergal.*

"Come unto Me
And I will give you rest." "Once more the voice
Is in my ear. It seems to echo now
The mournful hope that death should give me rest,
And yet I know this is no dream-like sound
Of sad Death making answer. It is the voice
Of life and not of death!" . . . . He spake
Of giving rest, and on the bitter cross
He gave the promised rest? O Christ the King!
We also wander on the desert hills,
Though haunted by Thy call, returning sweet
At morn and eve; we will not come to Thee
Till Thou hast nailed us to some bitter cross,
And made us look on Thine; and driven at last
To call on Thee with trembling and with tears—
Thou lookest down in love, upbraiding not,
And promising the Kingdom! —*B.M.*

### Reading.

Try to be in your own household what God is in the midst of His creation; the moving spirit, Himself immovable.

As opposed to passion, change, fitfulness, or laborious exertion, repose is the especial and separating characteristic of the eternal music and power. It is the "I am" of the Creator opposed to the "I become" of all creatures; it is the sign alike of the supreme knowledge which is incapable of surprise, the supreme power which is incapable of labour, the supreme volition which is incapable of change; it is the stillness of the beams of the eternal chambers, laid upon the variable waters of ministering creatures. . . . . So that the desire of rest planted in the heart is no sensual or unworthy one, but a longing for renovation.

The repose necessary to all beauty is repose not of inanition, not of luxury, nor of irresolution, but the repose of magnificent energy and being; in action the calmest of trust and determination: in rest, the consciousness of duty accomplished and of victory won; and this repose and this felicity can take place as well in the midst of trial and tempest as beside the waters of comfort; they perish only when the creature is unfaithful to itself.—*Ruskin.*

### Notes and Queries.

**The contents of the Editor's Letter-box nave now reached such unwieldy proportions that it has become necessary to establish a hard and fast rule regarding Answers to Correspondents. In future, all questions requiring replies will continue to be answered in this column without any fee. If an answer is required by letter, a fee of half-a-crown must be enclosed with the note containing the enquiry. We are always pleased to help our numerous correspondents to the fullest extent, and we can trust them to sympathise in the overwhelming amount of writing which makes the new rules a necessity.**

**Every communication must be accompanied by the writer's name and address, otherwise it will receive no attention.**

*Recovery of Fee.*

(155) Could you tell me what fee can be recovered by a maternity nurse when the patient is confined four weeks before the date on which the nurse is engaged?—*Ignorant.*

Arrangements for such a contingency ought to be made in writing at the time the engagement is accepted. It is customary for the nurse to receive half fees; but we are afraid that without a written agreement it will be difficult to recover it.

*Accounts.*

(156) Kindly tell me where I can get a good little book in which the matron of a small hospital can keep all her accounts, also the price of same.—*C. (Norfolk).*

We have asked the Manager of the Scientific Press to send you particulars.

*R.B.N.A.*

(157) Can you tell me how myself and several other nurses can become British Association nurses? We have three first-class certificates, also L.O.S. diplomas.—*M.H.*

Write to the Secretary, 17, Old Cavendish Street, W.

*Sterilising Milk.*

(158) 1. Can you give me rules for sterilising milk? Also, if a special apparatus is best, where can it be bought? 2. We think it would be an excellent thing for our tubercular patients to use paper handkerchiefs and burn them, so as to do away with that source of infection. We have heard there are such things made. Can you give us any information on the point?—*C. Jaggars.*

1. The rule for sterilising milk is simple. Immerse the vessel or bottle containing fresh milk (uncorked) in a pan of boiling water, which keep boiling, and after the milk has reached its highest temperature maintain it at that point for twenty minutes. The steriliser devised by Dr. Aymard, of Ipswich, is very good. G. Bennett, of Ipswich, is the manufacturer. 2. The Army and Navy Stores, 105, Victoria Street, Westminster, have paper squares for use either as handkerchiefs or table napkins. At Brompton all used handkerchiefs are burned every day, a rule that should always be observed with tubercular cases.

*"Materia Medica."*

(159) Would you kindly let me know through your "Notes and Queries" column the name or names of a reliable, up to-date "Materia Medica" and a "Practice of Medicine"?—*L. J. C.*

"Materia Medica and Therapeutics," Dr. Mitchell-Bruce (Cassell), and "Practice of Medicine," either by Dr. Taylor or Dr. Osler.

*Books.*

(160) Can you tell me of a book course of medical botany, one of medical Latin, and one which will furnish the right terms for medicines and the correct abbreviations of the same?—*Orthopædic.*

Possibly a good "Materia Medica," such as Dr. Mitchell-Bruce's, (Messrs. Cassells), will combine all the information you need.

*Training in Cape Town.*

(161) Will you kindly tell me in your inquiry column (1) whether there are any good hospitals in or near Cape Town; (2) if there would be good openings for trained or partially trained nurses; and (3) if the salaries of such are more than those given in English hospitals, or to private or district nurses working at home?—*B. C.*

1. There are very good hospitals in Cape Colony. The New Somerset Hospital, Cape Town, has a staff of 24 nurses, and the matron is Sister Alicia. 2. There are openings everywhere for well-trained nurses if they can afford to wait for them; but (3) abroad salaries are very much as at home, and the money does not go nearly so far.

*Maternity Case.*

(162) Will you kindly tell me if there is any free home for confinement case? Patient without friends or money, and has lost her husband. It is an unusually sad case.—*Nurse.*

The East-end Mothers' Home, 396, Commercial Road, admission free; the British Lying-in Hospital, Endell Street, W.C., admission by subscriber's letter; Queen Charlotte's Hospital, Marylebone Road, N.W., admission by subscriber's letter; the General Lying-in Hospital, York Road, Lambeth, S.E., admission by subscriber's letter. Write to the secretaries of the above for full particulars.

(163) "Sister Margaret" writes: Would you kindly give your valuable advice about the following? I am shortly going to India, and seriously think of being inoculated against typhoid. Are the majority of physicians for or against it? Hoping soon to see your reply.

Inoculation as a prophylactic against typhoid fever is a matter which is still *sub judice.* We do not think any means have been taken to ascertain the proportion of physicians who are in favour of the course, nor do we think that such matters are to be decided by majorities. So far as inoculation for typhoid fever has gone there seems no great objection to it, except the febrile reaction which it sets up, which is sometimes considerable. We would, however, strongly urge that no one should presume on the fact of having been inoculated, or let it tempt them to in any way relax the precautions against typhoid of which experience has taught the necessity.

### Travel Notes.

By Our Travelling Correspondent.

Rules in regard to Correspondence for this Section.—All questioners must use a pseudonym for publication, but the communication must also bear the writer's own name and address as well, which will be regarded as confidential. All such communications to be addressed "Travel Editor, 'Nursing Mirror,' 28, Southampton Street, Strand." No charge will be made for inserting and answering questions in the inquiry column, and all will be answered in rotation as space permits. If an answer by letter is required, a stamped and addressed envelope must be enclosed, together with 2s. 6d., which fee will be devoted to the objects of the "Hospital Convalescent Fund." Any inquiries reaching the office after Monday cannot be answered in "The Mirror" of the current week.

### VI.—MONTE CARLO AND MONACO.

There is a great deal more to tell you about the environs of Nice, but for variety we will now journey along the coast in the direction of Italy, and return to the exploration of the Chemin de fer du Sud later on. After passing Riquier, a suburb of Nice, we come to Villefranche. I shall pass over this for the present, as I want to give you an illustration of this earthly paradise, and I shall have no space for it this week. The next station is Beaulieu, where Lord Salisbury takes short rests from the cares of Government in his charming villa La Bastide. As I told you last week, round Beaulieu is the most sheltered spot on the Riviera, and many people prefer a residence there to one in Nice; it is much quieter, and the situation is romantically beautiful, but there are not the social attractions of Nice, nor its many conveniences. There are some good hotels, decidedly cheaper than those of Nice and Cannes; terms nominally from 12 to 20 francs.

### St. Jean.

There is a lovely walk—also practicable for a carriage, but the road is very narrow—to the Port of St. Jean. The whole of this little peninsular is exquisitely lovely; on all sides is unsurpassed beauty. Villefranche, on the one hand, and Cap Martin on the other, with Bordighera in the distance, and the rugged heights of Eze, Roquebrune, &c., topped by the Corniche, to the north, form a striking picture. St. Jean is the great place to eat bouillabaisse, of which we read in Thackeray and the immortal "Trilby."

### Caution to Cyclists.

I think I ought to warn all cyclists of the dangers to be encountered along these fascinating roads. Many of them are excellent, and cycling is in great favour, but it requires to be undertaken with immense care, even by the most experienced. The turns are so sharp and the precipices so terrific that no one, however reliable, should go beyond the most moderate pace anywhere along this coast. I am reminded to speak of this now by having in my recollection a truly awful corner of this sort between St. Jean and Beaulieu, where a little want of caution would inevitably lead to certain death in a most appalling way.

### "The Garden of Armida."

From Nice to Monte Carlo the train takes 45 minutes, a fairly leisurely pace certainly; but when it is along such a road one does not mind the time occupied. Every bend of the line discloses fresh beauties. I do not give you hotels at Monte Carlo, because, exquisite as is the spot in which what Symonds calls "the garden of Armida" is placed, it is not a suitable residence for invalids. The life is far too exciting and unrestful, and to a thoughtful mind cannot but have a painful effect; there seems such a cruel inconsistency in having so sinful a centre to one of the most exquisitely lovely spots in the whole world. Year after year goes by and the gaming tables are always thronged with a crowd, the most saddening in the world to witness, but never a season without suicides—unhappy creatures, driven by absolute despair to take the lives given them by God to be a blessing and a joy to others. All cases of suicide and violence of all sorts are carefully hushed up, lest harm should be done to this colossal enterprise, and if an unlucky gamester is entirely cleaned out of money and reputation, the proprietors of the Casino see that he has means to leave the place. I spent four hours one day watching the tables, and there is indeed a sinister fascination about it. Doubtless it is by no means avarice alone that makes gambling so irresistible to some temperaments; it is the extraordinary excitement attendant upon it, which, once allowed to take firm hold, never relaxes its grip upon its unhappy victim, until, more likely than not, he is beggared in pocket and character, and sinks to rise no more—one of the vast army of those "gone under." I could not decide which were the most distressing to watch, the very aged or the very young. On the whole, perhaps the former; to see an old man of 75 or 80, already in the fell grasp of palsy or paralysis, and whose thoughts ought to be directed beyond the world (which, in the natural order of events, he must so soon quit), watching the roulette wheel with frenzied eye, and raking in his gains as if they were an immortal crown of glory, is painful to the last degree. With the young we may at least hope that there will be time for repentance and amendment of life. No one under the age of 21 is admitted, nor any person commercially engaged in the town; there is much astuteness displayed in these regulations, for the ruin of a fellow townsman would act unfavourably on the Blanc establishment, and the suicide of a girl or boy in their teens would have similar results. Everything is free in Monte Carlo—the lovely gardens, the beautiful concerts, even the luxurious dressing rooms—all free. Everything is done to make the downward course smooth and easy, and alas! these efforts are only too thoroughly crowned with success.

### Monaco and the Jardin St. Martin.

Monaco is most singularly placed; it is almost an island, joined only to the main land by a narrow neck of rock. Here the Prince has his palace and his municipal buildings and his barracks for the army, consisting of about a dozen men; here, too, is the Cathedral, built a few years since on the site of the ancient chapel belonging to the Grimaldi family, which was a thirteenth-century building. The palace is not interesting, having been restored out of all originality. Monaco has a little station all to itself although so close to Monte Carlo; this causes some slight confusion to the minds of the uninitiated. I got into the train last year with an American party, all of us *en route* for Monte Carlo. The conduct of these excellent people almost drove the guard mad; they all bundled out with immense labour at Monaco, the carriage being, as usual in France, a kind of young Mont Blanc to ascend and descend, and papa and mamma were substantial. From their conversation I gathered that they wanted Monte Carlo, and asked them if it was so. "Of course. I guess this *is* Monte Carlo!" "No!" I replied, "this is Monaco, where the Prince's palace is; do you wish to see that or the gaming tables?" Papa was by this time ponderously remounting and gathering his family together—evidently he was bent upon trying his fortunes at *Rouge et Noir*. The guard wrathfully pushed behind as I endeavoured to explain to him the complications of the situation. We rolled on, and I tried to penetrate the dense fog which enshrouded their faculties, with the result that papa was righteously indignant to think after all they had missed Monaco, though I assured him that trains were numerous to and fro. On the reappearance of the guard at Monte Carlo, they harangued him with much fervour, and heaped reproaches both loud and deep on his devoted head. He snorted and glared defiance, being aware that something uncivil was being said, but happily not comprehending a word. Whether eventually they succeeded in seeing either place is a page of unwritten history. I think probably not, as, after the usual transatlantic manner, they had only allowed themselves one and a half hours to "do" both Monaco and Monte Carlo. Monaco is reached by a curious pitched pathway with broad shallow steps. As this road winds round the rock, round bastions are set at the angles. The effect is delightfully picturesque—the brilliant blue sea and sky, the romantic Port of Hercules at our feet crowded with fishing craft, the white walls and towers close at hand, crowned with the palace and cathedral, make a never-to-be-forgotten picture. Under the palace lies the Jardin St. Martin; here art has so far assisted Nature as to make it possible to walk along this Garden of Eden, where once an almost perpendicular wall of rock gave no foothold. The natural luxuriance of the spot has not been spoilt in any way. Aloes, cacti, prickly pears, cytisus, and scarlet geraniums grow richly all over the rocks, right down to the edge of the water. The last-mentioned attain an enormous height, six and eight feet high, and in situations where the foot of man has never trod the birds carry the seed, and they flourish untouched. Roses, too, are most prolific, and a beautiful purple creeper, the name of which I do not know. The surroundings are altogether so brilliant that the effect is that of fairyland. It is a delightful plan to take one's lunch in a basket and start for Monaco by the early train, spend the entire day in this garden of the gods, returning in the late afternoon. The sun and air cure cannot be carried out better than along these idyllic shores.

Nurses on the Riviera.

We have been asked to make known that the Elgin Nursing Institution has opened a branch at Alassio to be opened from November to May. Application to be made to Superintendent Miss Ellison, Châlet Santa Croce, Alassio, N. Italy. The beach is very fine, and, unlike most of those belonging to Riviera towns, is formed of fine sand. The little town has two distinct seasons, the winter one for foreigners and those who are delicate, and the summer for Italians who spend their villegiatura there. The bathing is excellent, the air warm and delightful, except when the wind is in the east, Alassio being exposed to that unkind blast. Visitors should adhere carefully to the rule recommended to all English residents of drinking only boiled water; not that the water of Alassio is specially to be distrusted, but because it is the only really safe plan abroad, and, indeed, it would be much wiser if people kept to the same excellent plan in England. The wine of the country is cheap and good, but somewhat limited in quantity. A good deal of Sardinian wine is drunk, which is also pleasant and wholesome. There is a small English colony, many villas being occupied year after year by the same families. Alassio is very accessible, being about one hour by rail from San Remo and two and a half from Genoa. The excursions in the neighbourhood are numerous, and the country, as almost everywhere on the Riviera, very beautiful.

Intending visitors may be glad to know that there is an English church and library, and this winter two long-felt wants have been supplied, viz., an institution where English-trained nurses and masseuses can be obtained and a tea garden. Albenga, close at hand, is a delightful resort for artists. There are several good hotels in Alassio. Hotel Suisse is near the station, and the Grand Hotel near to the sea. Pensionnaires are also taken at the Villa Garibaldi. Dr. Boon is the English doctor resident in Alassio, and a few delicate people can be received in the Elgin Nursing Home. Another advantage to be obtained from this institution is that the services of a daily nurse for visits and occasional night work may be had by arrangement.

### TRAVEL NOTES AND QUERIES.

Rome (Novice).—The season may be said to last from October to March. It is delightful during any of those months. The water is very good, the cold not so searching as in Florence.

### Everybody's Opinion.

[Correspondence on all subjects is invited, but we cannot in any way be responsible for the opinions expressed by our correspondents. No communication can be entertained if the name and address of the correspondent is not given, as a guarantee of good faith but not necessarily for publication, or unless one side of the paper only is written on.]

### OUR CLOTHING DISTRIBUTION.

The Resident Lady Superintendent, East End Mothers' Home, London, E., writes: Thank you very much for your gift of clothing received safely. We are indeed grateful for all your repeated kindness to us.

The Lady Superintendent, West Ham Hospital, Stratford, London, E., writes: I have to thank you for a parcel of garments sent to us for our patients. They reached us on Saturday last. They gave much pleasure and comfort to some of the patients. I need hardly tell you how poor a class of patients we get in this hospital and district.

### HOME FOR NURSES NO LONGER ABLE TO WORK.

"A Constant Reader" writes: Through the medium of your valuable paper I would like to thank Miss B. C. Davis for her very sensible letter on the above. She has put into words what has been in my thoughts for a considerable time. Such a home would not only be a boon, it is an absolute necessity, and were the scheme taken in hand by a few influential and business-like people nurses themselves would surely give all the help in their power. Besides, in time, why should it not be almost—if not quite—supported by the nursing profession? It will be said, "But if nurses would join the National Pension Fund how much worry and trouble as to their future would be saved them." My reply is that it is impossible for a great number to do so, owing to age or other reasons, the premiums being too high. It is appalling to think that after a nurse has given the best years of her life to the work she has simply nothing in the shape of home comforts to look forward to, either because she has no relatives to whom she can apply for help, or because she is too independent to ask for assistance. I trust Miss Davis's appeal will not be allowed to drop, for a more needed institution has never been pleaded for.

⁂ The above letter shows the absolute need of the Royal National Pension Fund for Nurses, which provides in a more liberal way than any other scheme for the old age of nurses. In the first place, a "home" ties a number of nurses to one locality irrespective of its desirability as regards the health, convenience, or wishes of each inmate; in the second, it forces a number of elderly women, already set, by force of circumstances, in the same groove, into so close a companionship that it could not fail to be irksome, even to the loftiest natures. It is far removed from the ideal life—that is, family life—in which men and women of different ages and various pursuits bring each his or her quota of interest to each individual. A nurse with even a small pension can choose within very wide limits where she will live, with what kind of people she will live, and with what interests she will occupy herself. Such liberty is worth a struggle.—Ed. *T. H.*

### Presentations.

Miss Henriette Leslie was presented on the 6th inst. with a handsome silver travelling clock on the occasion of her resigning her post as matron of the private nurses' home attached to the Preston and County of Lancaster Royal Infirmary. The Chairman of the Board of Management, in making the presentation, expressed the appreciation in which Miss Leslie's services were held. They have extended over a period of sixteen years, during the last eight of which she has been in the position from which she now retires.

